# Improving and Optimizing Mechanical Properties of Glass Fiber-Reinforced Composites via Geometric Optimization of Nanofillers Using Co-Curing Processes

**DOI:** 10.3390/polym18060777

**Published:** 2026-03-23

**Authors:** Eonsu Yun, Hyunjong Choi, Joon Seok Lee, Byoung-Sun Lee, Hyunchul Ahn

**Affiliations:** 1 Department of Fiber System Engineering, College of Engineering, Yeungnam University, Gyeongsan 38541, Republic of Korea; yys2394@ynu.ac.kr (E.Y.); wldwld0802@yu.ac.kr (H.C.); leejs@ynu.ac.kr (J.S.L.); 2 Department of Fiber System Engineering, Dankook University, Yongin 16890, Republic of Korea

**Keywords:** glass fiber-reinforced composites, co-curing, adhesion, nanoparticle, graphene nanoplatelets

## Abstract

This study investigates the effects of the co-curing process and nanoparticle reinforcement on the mechanical performance of plain-woven glass fiber-reinforced plastic (GFRP) adhesive joints, aiming to address the limitations of traditional fastening methods and the inherent brittleness of epoxy adhesives. Specifically, spherical silica (SiO_2_) and plate-like graphene nanoplatelets (GNPs) were incorporated into the epoxy matrix at varying concentrations (0.25 to 1.0 wt.%) to evaluate the influence of particle geometry on joint integrity. Experimental results demonstrated that the co-curing technique yields superior mechanical properties compared to secondary bonding, exhibiting improvements of 35% in shear strength (from 10.97 MPa to 14.83 MPa) and 12% in flexural strength (from 72.57 MPa to 81.28 MPa) due to enhanced chemical interlocking. Furthermore, the addition of nanoparticles significantly improved joint performance, with the optimal content identified at 0.75 wt.% for both particle types. Notably, GNPs outperformed SiO_2_, enhancing shear and flexural strengths compared to the neat co-cured baseline. Ultimately, the 0.75 wt.% GNP-reinforced material exhibited a shear strength of 21.22 MPa and a flexural strength of 104.09 MPa. Morphological analysis revealed that while SiO_2_ contributes to reinforcement primarily via crack deflection, the high-aspect-ratio GNPs provide superior energy dissipation through crack bridging and pull-out mechanisms. Consequently, this study suggests that the co-curing process combined with an optimal concentration of GNPs presents a highly effective strategy for maximizing the reliability and structural efficiency of composite joints in weight-critical applications.

## 1. Introduction

Recently, fiber-reinforced plastics (FRPs), particularly glass fiber-reinforced plastics (GFRPs), have established themselves as pivotal lightweight materials replacing traditional metals such as steel and aluminum. This shift is driven by their high specific strength, specific stiffness, and superior corrosion resistance, making them essential in aerospace, automotive, marine, and defense applications [1,2]. The fabrication of large-scale composite structures inevitably necessitates the joining of individual components. However, traditional mechanical fastening methods utilizing rivets or bolts serve as primary sources of structural degradation in composites. Specifically, drilling holes for fastening in FRPs induces fiber discontinuity and stress concentration, thereby shortening the fatigue life of the structure. Furthermore, it precipitates damage such as fiber peeling [3], delamination [4], and micro-cracking [5] at the fastening sites [6,7]. Additionally, the inclusion of fasteners diminishes the weight-saving benefits and poses risks of galvanic corrosion between dissimilar materials, consequently limiting the full exploitation of the inherent advantages of composite materials [8].

To address these challenges, adhesive bonding has garnered significant attention as a viable alternative. Adhesive bonding offers distinct advantages, including the mitigation of stress concentrations by distributing stress over a larger area, as well as enhancements in airtightness and fatigue resistance [9]. Bonding techniques are generally classified into secondary bonding (SB), co-bonding (CB), and co-curing (CC) [10]. Among these, the co-curing process, which involves the simultaneous curing of the composite laminate and the adhesive, provides exceptional process efficiency by eliminating the surface preparation and additional curing steps required in secondary bonding [11,12]. More importantly, co-curing exhibits superior characteristics in terms of both mechanical and chemical interlocking compared to secondary bonding. While secondary bonding relies primarily on mechanical interlocking by applying adhesive to an already cured surface, co-curing facilitates the active inter-diffusion of polymer chains as the semi-cured composite and adhesive cure simultaneously. This mechanism eliminates distinct interfaces between the laminate and the adhesive [13], fostering robust chemical bonding that effectively suppresses interfacial delamination [14]. recent studies have explored advanced materials to further enhance the performance of co-cured joints. For instance, Quan et al. [15] proposed a novel co-curing bonding strategy for carbon fiber/epoxy composites by replacing traditional aerospace epoxy adhesives with surface-treated carbon fiber/PEEK (CF/PEEK) tapes. Their investigation demonstrated that under optimized thermal conditions, the CF/PEEK co-cured joints exhibited remarkable improvements in lap-shear strength, fatigue life, and Mode-I/Mode-II fracture toughness at both ambient and elevated temperatures. This significant enhancement in structural stability was primarily attributed to the massive plastic deformation of the PEEK resin and the extensive interlocking mechanisms formed during the co-curing process.

Despite the efficiency of the bonding process, epoxy resins, which are widely employed as adhesives and matrices, suffer from inherent brittleness and low fracture toughness [16]. These deficiencies can lead to failure of the joint under impact or shear loading, thereby compromising structural reliability. To overcome these limitations, extensive research has focused on nanocomposites, where nanoparticles such as graphene [17], carbon nanotubes (CNTs) [18], silica (SiO_2_) [19], and alumina (Al_2_O_3_) [20] are dispersed within the epoxy matrix to enhance mechanical properties [21]. The reinforcement mechanism is governed not only by the type and content of the particles but also by their geometric morphology. For instance, spherical particles like silica and alumina are effective in relieving stress concentrations and deflecting crack propagation paths [22]. Conversely, anisotropic particles with high aspect ratios, such as CNTs or graphene [23], can dissipate higher fracture energy through bridging and pull-out effects across crack surfaces [24,25]. However, these high-aspect-ratio particles possess a significantly larger specific surface area compared to spherical particles, which can cause a dramatic increase in resin viscosity even at low loading levels. Excessive viscosity hinders resin flow during the co-curing process and may induce void formation; thus, an optimal design that balances the reinforcement effect with processability is imperative [26]. While existing studies have largely focused on optimizing the content of single particle types, there is a lack of comparative analysis regarding how particles with distinct geometries influence stress transfer and fracture behavior within the adhesive. In particular, considering the complex loading conditions of real-world structures, it is critical to analyze the impact of nanoparticle geometry on flexural strength under bending moments, in addition to simple shear strength, to ensure joint reliability.

In this study, spherical silica nanoparticles and two-dimensional GNPs were selected to systematically evaluate the influence of morphological differences on the rheological properties of the resin. Subsequently, the correlation between these properties and the shear and flexural strengths of CC GFRP joints. Through this approach, the reinforcement mechanisms dictated by nanoparticle shape were compared, the optimal particle type and content conditions for the co-curing process were identified, and these findings were verified through comprehensive mechanical property analysis.

## 2. Materials and Methods

### 2.1. Materials

In this study, a plain weave glass fiber fabric (WR570-100, Owens Corning, Toleldo, OH, USA) was employed as the primary reinforcement for the composite laminates. The mechanical properties of the glass fiber are summarized in Table 1. A Bisphenol-A epoxy resin (YD-128, Kukdo Chemical, Seoul, Republic of Korea) and a curing agent (H-4065, Kukdo Chemical, Seoul, Republic of Korea) were selected for the matrix system. The resin and hardener were mixed at a weight ratio of 4:1 to serve as both the structural matrix and the adhesive.

To enhance the mechanical properties of the adhesive, spherical silica (SiO_2_, SO-C6, Admatechs, Gifu, Japan) and GNPs (<2 μm particle size, Sigma-Aldrich, St. Louis, MO, USA) were utilized as nanofillers. The detailed physical characteristics of these nanoparticles are presented in Table 2. To evaluate the mechanical performance relative to the bonding method, composite joints were fabricated using both secondary bonding (SB) and co-curing (CC) techniques. It should be noted that the nanofillers were exclusively incorporated into the adhesive layer of the CC specimens to investigate the reinforcement effect in the co-curing process.

### 2.2. Manufacturing Methods

Ultrasonic dispersion was employed to uniformly disperse nanoparticles into the epoxy resin at various weight fractions (0.25, 0.5, 0.75, and 1.0 wt.%). First, the nanoparticles were mixed into 200 mL of acetone and subjected to ultrasonication for 1 h. To prevent excessive temperature rise due to ultrasonic energy and rapid evaporation of acetone, the dispersion process was conducted in an ice bath. Subsequently, 100 g of epoxy resin was added to the nanoparticle-dispersed acetone solution. The mixture was then stirred using a mechanical stirrer on a hot plate maintained at 100 °C for 2 h. This high-temperature stirring process facilitated the mixing of the epoxy and nanoparticles while simultaneously ensuring the evaporation of the acetone.

**Figure 1:**
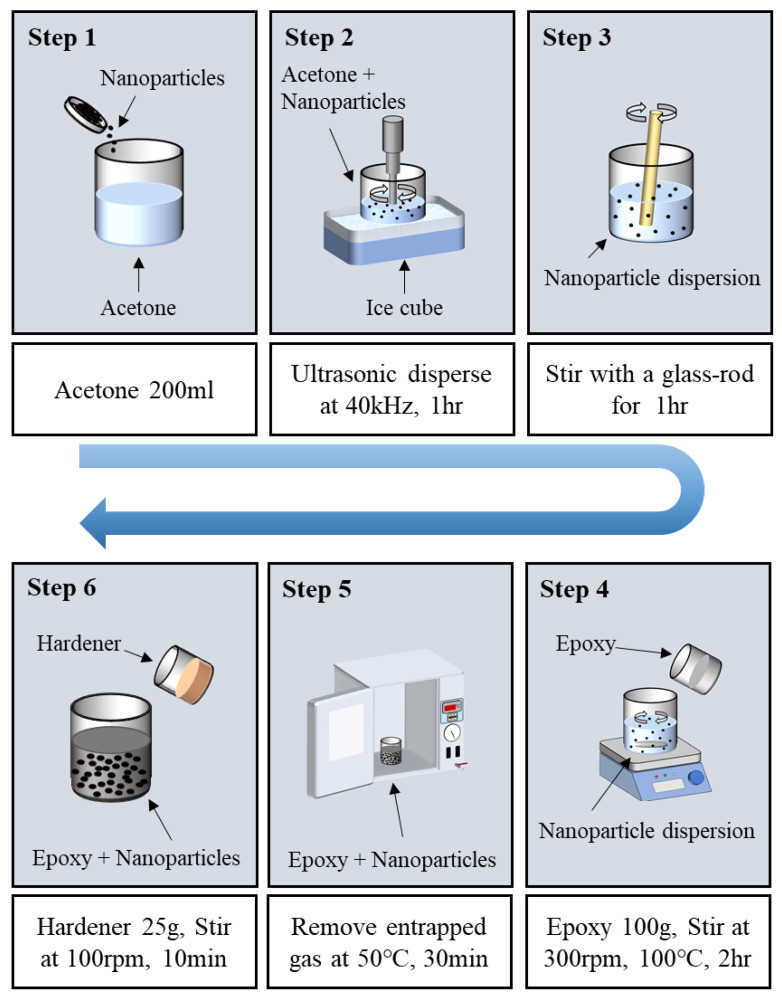
Schematic diagram of enhanced resin preparation.

To eliminate residual acetone and air generated during mixing, the mixture was placed in a vacuum oven and degassed at 50 °C for 30 min. Finally, 25 g of the curing agent was added to the degassed mixture and stirred at 100 rpm for 10 min at room temperature using a mechanical stirrer to obtain the final nanoparticle-reinforced epoxy adhesive. The overall dispersion and preparation process is illustrated in Figure 1.

**Figure 2:**
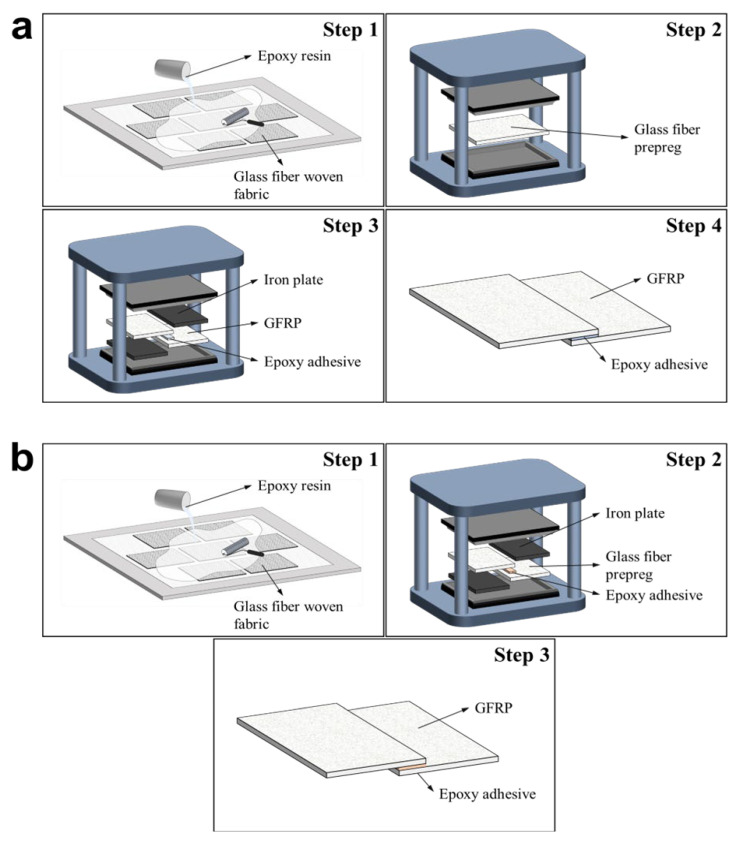
Schematic diagram of (**a**) the secondary bonding process and (**b**) the co-curing process.

Composite joints were fabricated using the prepared resins via hand lay-up and compression molding techniques. For the SB specimens, pure epoxy resin without nanoparticles was used to establish a baseline for mechanical property comparison. Two GFRP laminates were initially fabricated, each consisting of 7 plies of glass fiber fabric impregnated with neat epoxy resin via the hand lay-up method. The impregnated laminates were fully cured under a pressure of 5 bar (0.5 MPa) at 80 °C for 30 min. After curing, the laminates were cooled and demolded once the temperature reached under 50 °C. The cured GFRP laminates were then cut to size for the secondary bonding process. During bonding, the GFRP plates were placed in a mold along with a 3 mm steel plate serving as a spacer. Neat epoxy resin was applied to the joint interface as an adhesive, followed by a second curing and demolding cycle under the same conditions as the initial fabrication. The overall secondary bonding process is depicted in Figure 2a. The CC specimens were also fabricated using 7 plies of glass fiber fabric via the hand lay-up method. Similar to the SB process, a steel plate spacer was positioned within the mold. However, to enhance bonding performance, the previously prepared nanoparticle-reinforced epoxy adhesive was applied between the joint interfaces of the two uncured preforms. Subsequently, a co-curing process was performed to cure both the entire laminate stack and the adhesive simultaneously. The curing conditions were identical to those used for the SB samples. The overall co-curing fabrication process is illustrated in Figure 2b. Each composite panel was fabricated with dimensions of 295 × 245 × 2.5 mm, and the final test specimens were subsequently cut to a width of 25 mm using a waterjet. The bonded overlap area was 25 × 25 mm, and the adhesive bondline thickness was uniformly maintained at 0.5 mm using a 3 mm spacer. A total of 10 types of specimens were fabricated. The designations and detailed conditions for each sample are summarized in Table 3. To ensure the statistical significance and reliability of the mechanical test results, a minimum of five identical specimens were tested for each experimental condition. All reported data represent the average values, with standard deviations plotted as error bars in the corresponding figures.

### 2.3. Experimental and Analysis

Rheological analysis was conducted to evaluate the processability of the nanoparticle-reinforced epoxy adhesives. The viscosity of the prepared adhesive resins was measured using a rheometer (MCR 302e, Anton Paar, Graz, Austria). A parallel plate geometry was employed, and to simulate actual molding conditions, the measurement was performed under isothermal conditions at 80 °C, identical to the processing temperature.

To assess the effect of nanoparticle-reinforced epoxy adhesives on the mechanical performance of CC joints, specifically shear and flexural strengths, mechanical tests were performed using a universal testing machine (UTM, Instron 68FM-100, Instron, Norwood, MA, USA). First, single lap joint (SLJ) specimens were fabricated in accordance with ASTM D5868 standard [27] to measure shear strength. The shear tests were conducted at a crosshead speed of 13 mm/min until specimen failure occurred. Three-point bending tests were carried out to evaluate the flexural characteristics of the SLJs, following the ASTM D790 standard [28]. The same UTM was utilized, equipped with a three-point bending fixture. The dimensions of the flexural specimens adhered to ASTM D790 specifications, and the support span was set to 88 mm. To ensure the horizontal alignment of the specimen during testing, tabs were attached to the ends. The load was applied at a rate of 2.4 mm/min until failure.

Following the mechanical testing, the fracture behavior of the specimens was examined. The micro-morphology of the fracture surfaces and the dispersion state of the nanoparticles within the epoxy adhesive were investigated using field-emission scanning electron microscopy (FE-SEM, SU8600, Hitachi, Tokyo, Japan). Prior to observation, the specimens were sputter-coated with platinum to prevent surface charging and ensure conductivity.

## 3. Results and Discussion

### 3.1. Nanoparticle Enhanced Resin

**Figure 3:**
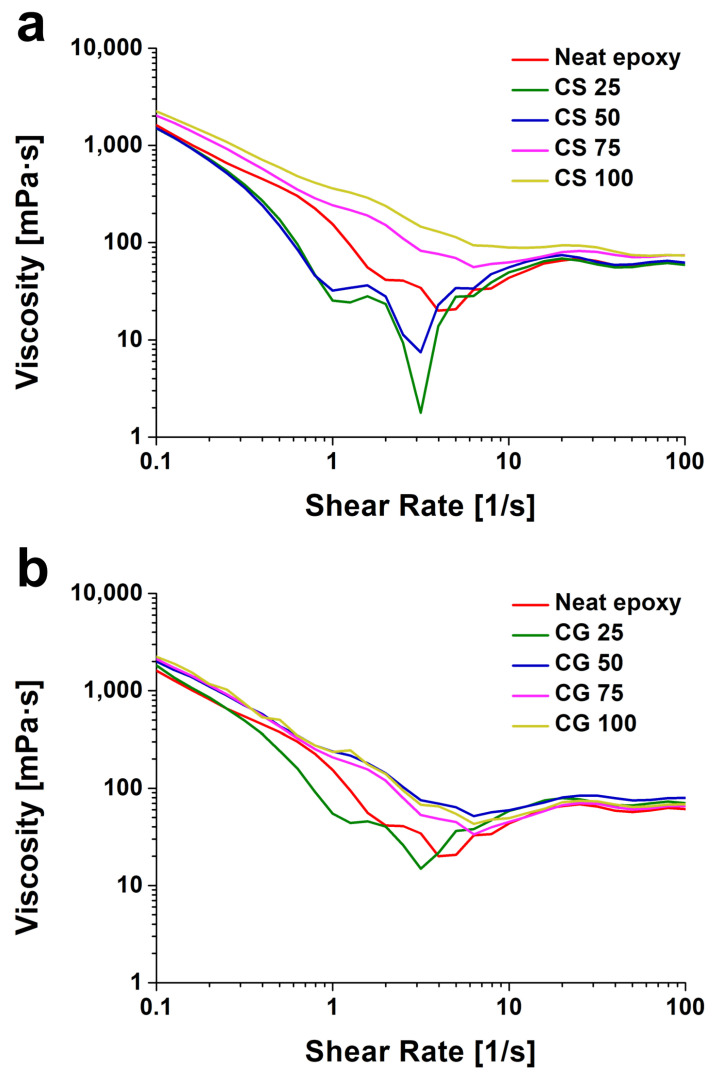
Viscosity of epoxy resin with different nanoparticle contents. (**a**) SiO_2_ enhanced resin and (**b**) GNPs enhanced resin.

Figure 3 shows the shear viscosity behavior of epoxy adhesives with varying types and contents of nanoparticles. First, regarding the initial viscosity, the neat epoxy exhibited a viscosity of 1613 mPa·s. In the case of SiO_2_-reinforced specimens (CS series), a decrease in viscosity compared to the neat epoxy was observed at low loading levels; the viscosities of CS 25 (0.25 wt.%) and CS 50 (0.5 wt.%) were measured at 1500 mPa·s and 1509 mPa·s, respectively. This reduction suggests that at low loading levels, the well-dispersed spherical SiO_2_ nanoparticles position themselves within the free volume between the epoxy polymer chains. Rather than forming a flow-restricting network, these rigid, smooth spheres rotate under shear stress and facilitate the sliding of adjacent polymer chains past one another. This phenomenon, commonly referred to as the “ball bearing” effect, effectively decreases the local entanglement density and mitigates intermolecular friction, thereby lowering the macroscopic viscosity of the resin system [29,30]. However, as the content increased, the viscosity rose to 2022 mPa·s for CS 75 and reached 2250 mPa·s for CS 100. This increase in the high-content regime is attributed to enhanced particle-particle interactions and agglomeration, which significantly increased flow resistance.

In contrast, the GNPs-reinforced specimens (CG series) displayed a consistent trend of increasing viscosity with higher filler content. Even at the lowest content, CG 25 showed an increased viscosity of 1819 mPa·s. This trend continued with CG 50 (2012 mPa·s), CG 75 (2115 mPa·s), and CG 100 reaching 2241 mPa·s. This behavior is ascribed to the high aspect ratio of the two-dimensional plate-like structure of GNPs. The large surface area of GNPs physically obstructs the movement of polymer chains, facilitating the formation of a rheological network even at low loadings [31].

Interestingly, at the highest loading condition (1.0 wt.%), both CS 100 and CG 100 exhibited similar viscosity levels. This implies that beyond a critical concentration, the particle content exerts a more dominant influence on viscosity increase than particle morphology. Furthermore, shear-thinning behavior, where viscosity decreases with increasing shear rate, was observed across all conditions. This phenomenon is likely due to the alignment of dispersed nanoparticles along the flow direction under applied shear stress, which reduces flow resistance. In conclusion, while variations in initial viscosity were observed with increasing particle content, even the maximum viscosities recorded for CS 100 and CG 100 did not show a deviation significant enough to compromise processability compared to the neat epoxy. This confirms that the influence of nanoparticles on the manufacturing process is negligible. Consequently, this study can validly isolate and examine the changes in interfacial properties induced by nanoparticle addition, independent of processing defects.

### 3.2. Bonded Composites

**Figure 4:**
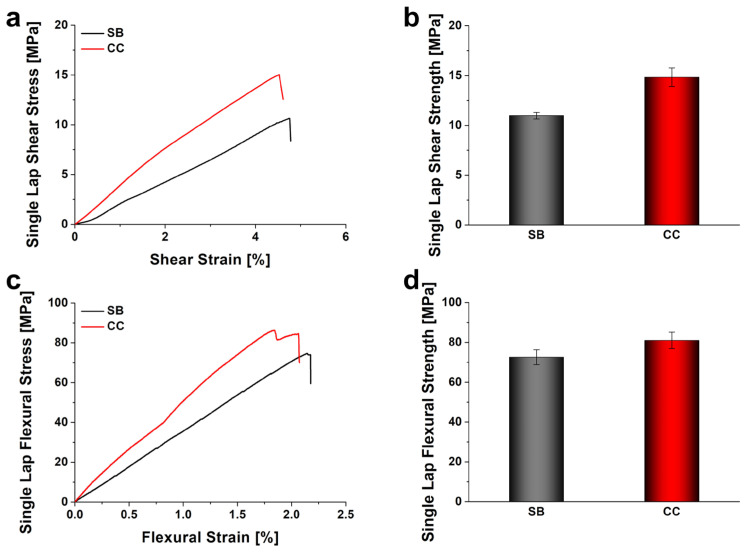
Effect of fabrication process on the mechanical properties of neat epoxy. (**a**) Shear stress–strain curves, (**b**) shear strength comparison, (**c**) flexural stress–strain curves, and (**d**) flexural strength comparison.

Figure 4 presents a comparative analysis of the shear and flexural strengths of SLJs fabricated via SB and CC processes. The experimental results demonstrated that the CC process yielded superior mechanical properties compared to SB across both shear and flexural modes. As shown in Figure 4a,b, the SB specimen recorded a shear strength of 10.97 MPa, whereas the CC specimen exhibited 14.83 MPa, indicating an improvement of approximately 35%. A similar trend was observed in flexural strength (Figure 4c,d); the SB specimen showed 72.57 MPa, while the CC specimen reached 81.28 MPa, reflecting an increase of about 12%. This discrepancy in strength is attributed to the mechanism of interface formation. While SB relies primarily on mechanical interlocking with a physically distinct interface, the CC process facilitates the formation of robust chemical bonds between the laminate and the adhesive as the matrix and adhesive cure simultaneously. This integration effectively unifies the layers, thereby enhancing resistance not only to shear loads but also to delamination induced by bending loads.

**Figure 5:**
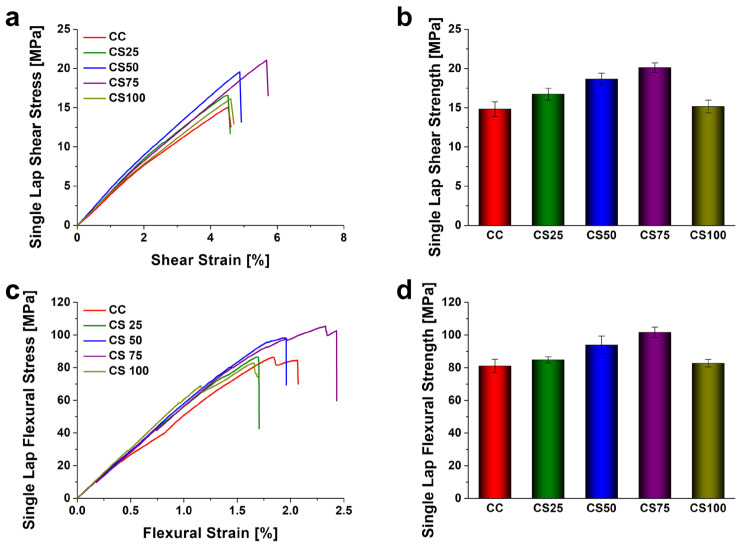
Effect of SiO_2_ nanoparticle contents on the mechanical properties. (**a**) Shear stress–strain curves, (**b**) shear strength comparison, (**c**) flexural stress–strain curves, and (**d**) flexural strength comparison. Note: Nanoparticle concentrations (wt.%) are calculated based on the total weight of the epoxy matrix and error bars indicating the standard deviation.

Figure 5 shows the effect of varying contents of SiO_2_ and GNPs on the shear and flexural strengths of SLJs. Regarding the SiO_2_-reinforced specimens, the shear strength increased progressively with particle content: 16.74 MPa for CS 25, 18.67 MPa for CS 50, and peaking at 20.12 MPa for CS 75, before declining to 15.16 MPa for CS 100. Flexural properties followed an identical trend, recording 84.80 MPa (CS 25), 93.94 MPa (CS 50), 101.67 MPa (CS 75), and decreasing to 82.77 MPa (CS 100). Notably, the CS 75 specimen (0.75 wt.%) exhibited the highest performance, showing improvements in shear strength of approximately 83% and 36%, and in flexural strength of approximately 40% and 25%, compared to the neat SB and CC specimens, respectively. However, at 1.0 wt.% loading, the strength decreased significantly, falling to levels comparable to the neat CC specimen, indicating a deterioration in properties beyond the optimal content.

**Figure 6:**
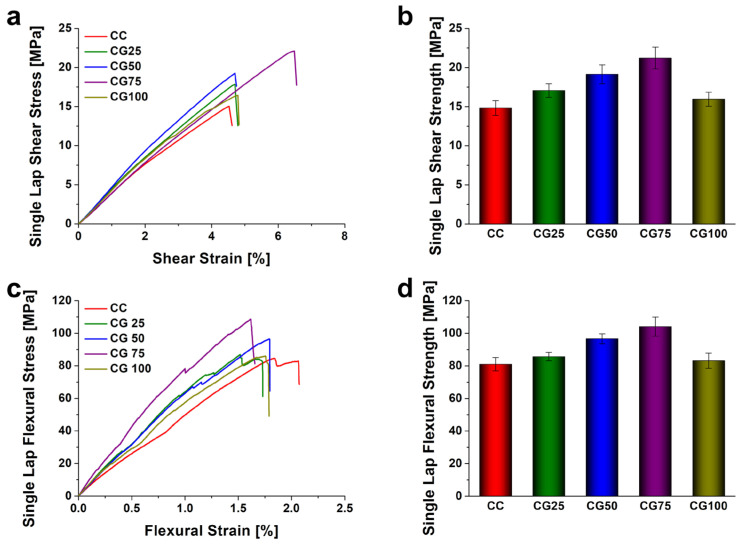
Effect of GNPs contents on the mechanical properties. (**a**) Shear stress–strain curves, (**b**) shear strength comparison, (**c**) flexural stress–strain curves, and (**d**) flexural strength comparison. Note: Nanoparticle concentrations (wt.%) are calculated based on the total weight of the epoxy matrix and error bars indicating the standard deviation.

Similar behavior was observed in the GNPs-reinforced specimens, as shown in Figure 6, but with a more pronounced reinforcement effect compared to the SiO_2_ series. The shear strengths were measured at 17.07 MPa (CG 25), 19.14 MPa (CG 50), 21.22 MPa (CG 75), and 15.96 MPa (CG 100). Likewise, flexural strengths were 85.65 MPa (CG 25), 96.67 MPa (CG 50), 104.09 MPa (CG 75), and 83.15 MPa (CG 100). The CG 75 specimen demonstrated the highest mechanical properties among all tested conditions, with improvements of approximately 93% and 43% in shear strength, and 43% and 28% in flexural strength, relative to the SB and CC baselines, respectively. Moreover, it outperformed the CS 75 specimen at the same loading level. However, similar to the CS series, the mechanical properties of the CG series degraded at 1.0 wt.% loading due to excessive particle addition, reverting to levels similar to the neat CC specimen.

The observed enhancement in strength with nanoparticle addition is attributed to the uniform dispersion of particles within the epoxy matrix, which increases stiffness and facilitates effective load transfer. Furthermore, the nanoparticles likely enhanced the toughness of the epoxy resin by delaying fracture through crack deflection and crack bridging mechanisms. Specifically, the incorporation of nanoparticles effectively delays macroscopic fracture by increasing the energy required for crack propagation. When an advancing crack front encounters the rigid nanoparticles, mechanisms such as crack deflection increase the total fracture surface area. Concurrently, the high-aspect-ratio GNPs bridge the crack wake and pull out from the matrix, transferring stress and dissipating significant strain energy. This localized energy dissipation restricts rapid crack growth, thereby transforming the typical brittle failure of the epoxy into a more ductile, tough fracture behavior. Conversely, the degradation in strength at 1.0 wt.% is ascribed to particle agglomeration. Agglomerated particles act as stress concentration points, initiating early failure, and hinder resin flow, potentially leading to defect formation such as voids at the joint interface [32,33]. Consequently, the optimal content for the nanoparticle-reinforced epoxy adhesive in this study was identified as 0.75 wt.%, regardless of particle type. In the comparison between particle types, GNPs outperformed SiO_2_ nanoparticles. This superiority is likely due to the high specific surface area and two-dimensional plate-like structure of GNPs, which are structurally more advantageous for resisting shear and bending loads.

**Figure 7:**
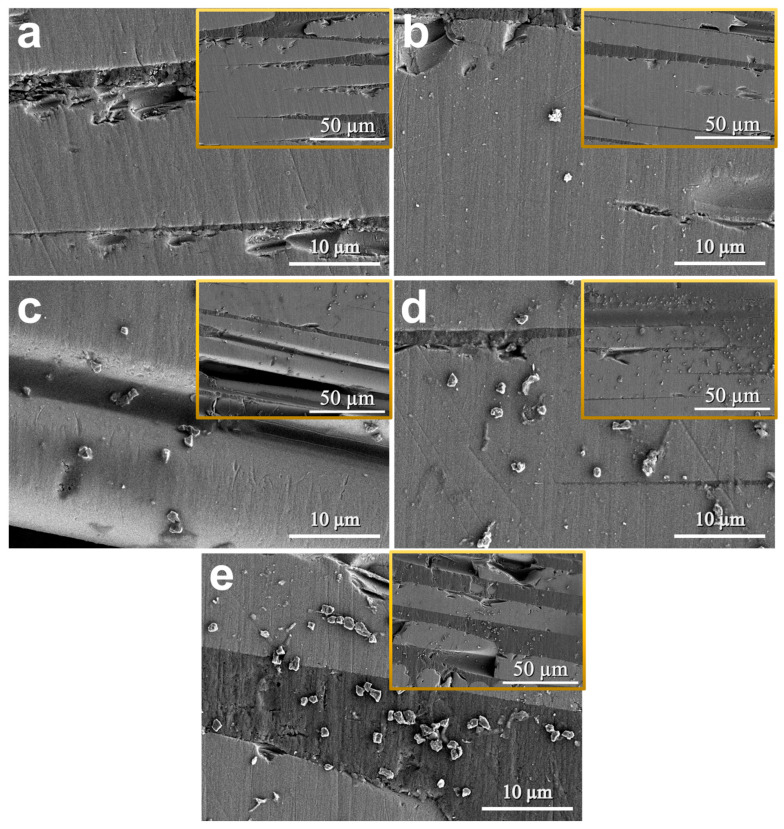
Fracture surface morphologies of the CS series. (**a**) Neat epoxy, (**b**) CS 25, (**c**) CS 50, (**d**) CS 75, and (**e**) CS 100. The magnifications of the main images and insets are ×3.0 k and ×1.0 k.

Figure 7 shows the SEM images of the fracture surfaces of pure epoxy and SiO_2_-reinforced epoxy adhesives. The morphological changes in the fracture surfaces were primarily analyzed to assess the dispersion and agglomeration status of the additives, and they showed a strong correlation with the strength enhancements observed in the mechanical tests. The fracture surface of the neat epoxy (Figure 7a) appeared generally smooth and flat, indicating rapid, linear crack propagation typical of brittle fracture behavior due to the absence of obstacles. In contrast, for the SiO_2_-reinforced specimens (Figure 7b–e), the surface roughness increased significantly with increasing particle content. Although the current magnification limits the direct observation of nanoscale crack interactions, this suggests that the dispersed SiO_2_ particles induced a crack deflection mechanism, twisting or diverting the crack path. This process elongates the crack propagation path and generates new surfaces, thereby effectively dissipating fracture energy and enhancing the toughness and strength of the adhesive. Specifically, the fracture surface of the CS 75 specimen (Figure 7d) exhibited the roughest and most complex morphology with uniform particle dispersion, confirming it as the optimal dispersion state for maximizing reinforcement while minimizing stress concentration. However, in the CS 100 specimen (Figure 7e), despite high roughness, distinct particle agglomeration was observed. These agglomerates likely acted as stress concentrators or weakened interfacial bonding, leading to premature failure, which explains the reduction in mechanical strength compared to CS 75.

**Figure 8:**
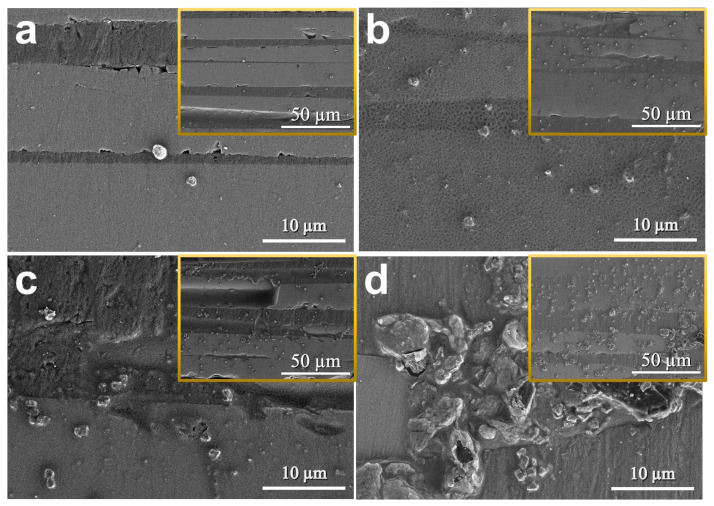
Fracture surface morphologies of the CG series. (**a**) CG 25, (**b**) CG 50, (**c**) CG 75, and (**d**) CG 100. The magnifications of the main images and insets are ×3.0 k and ×1.0 k.

Figure 8 shows the SEM micrographs of GNPs-reinforced epoxy adhesives. In contrast to the smooth fracture surface of neat epoxy, all GNPs-reinforced specimens exhibited highly rough and complex fracture surfaces. The primary reinforcement mechanism in the CG series is attributed to the intrinsic two-dimensional plate-like structure and high aspect ratio of GNPs [34]. The optimal balance between dispersion and reinforcement was achieved at 0.75 wt.%, maximizing efficient energy dissipation mechanisms. This structural advantage supports the superior mechanical performance of the CG 75 specimen. The reinforcement by GNPs is primarily driven by a combination of crack bridging and particle pull-out mechanisms. Specifically, while difficult to precisely resolve individual particle interactions at this magnification, the overall topography implies that the broad, plate-like GNPs bridged crack surfaces to physically inhibit growth and dissipated frictional energy during pull-out or interfacial debonding [16]. This resistance forced cracks to follow complex paths around the particles rather than propagating linearly, significantly improving fracture toughness.

The reason the CG series exhibited superior reinforcement compared to the CS series lies in these geometric differences. While spherical SiO_2_ particles mainly rely on crack deflection, the two-dimensional GNPs provide a much larger specific surface area, maximizing contact with the matrix. This not only enables more efficient stress transfer but also activates robust crack bridging mechanisms, dissipating significantly more fracture energy than simple deflection. However, in the CG 100 specimen (Figure 8d), substantial local agglomeration of GNPs was evident. Although the structural benefits of GNPs remain, these large agglomerates acted as structural defects within the matrix, serving as stress concentration points under load and limiting further strength enhancement at the 1.0 wt.% level, which directly corresponds to the decline in mechanical properties observed in the test results.

## 4. Conclusions

In this study, the synergistic effects of the CC process and nanoparticle reinforcement on the mechanical performance of GFRP adhesive joints were investigated. By comparing SB and CC methods, and by incorporating nanoparticles with distinct geometries (SiO_2_ and GNPs), the following key conclusions are drawn. The CC technique demonstrated significantly superior mechanical properties compared to the conventional SB process. The integrated curing process enhanced interfacial interlocking, yielding improvements of 35% in shear strength and 12% in flexural strength for the baseline joints. Regardless of the particle geometry, the mechanical strength of the reinforced joints consistently peaked at an optimal content of 0.75 wt.%. At this concentration, the nanoparticles were uniformly dispersed, effectively delaying fracture without the severe agglomeration observed at higher loading levels. At the optimal concentration (0.75 wt.%), the addition of GNPs enhanced the shear and flexural strengths by approximately 43% and 28%, respectively, compared to the neat CC baseline. In contrast, SiO_2_ yielded improvements of 35% and 25%. Morphological analysis confirmed that the high-aspect-ratio GNPs provided higher reinforcement efficiency and energy dissipation than the spherical SiO_2_ particles, leading to a distinct transition in the fracture mode. These substantial improvements in joint strength and toughness offer critical advantages for engineering applications. The combined strategy of the CC process and GNP reinforcement facilitates significant weight reduction by replacing traditional heavy mechanical fasteners. Furthermore, the enhanced stress distribution inherently improves safety factors and structural durability. Consequently, this approach presents a highly effective and promising solution for maximizing the structural integrity and reliability of composite joints in weight-critical applications.

## Figures and Tables

**Table 1:** Material properties of glass fiber woven fabric.

Tensile Strength (MPa)	Tensile Modulus (GPa)	Elongation at Break (%)	Warp Density (Ends/In)	Weft Density (Ends/In)	Areal Weight (g/m^2^)
3455	72.3	4.8	6.3	6.3	570

**Table 2:** Properties of nanoparticles.

Particle Type	Quality Level	Size (μm)	Surface Area (m^2^/g)	Bulk Density (g/m^2^)
SiO_2_	>99.8	1.8–2.3	1.5–2.5	-
GNPs	>99.5	<2	750	0.2–0.4

**Table 3:** Sample preparation according to nanoparticle contents and fabrication processes.

Name	Bonding Process	Reinforcement	Reinforcement Weight Fraction (wt.%)
SB	Secondary bonding	-	-
CC			
CS25		SiO_2_	0.25
CS50			0.50
CS75			0.75
CS100	Co-curing		1.00
CG25		GNPs	0.25
CG50			0.50
CG75			0.75
CG100			1.00

## Data Availability

The data presented in this study are available on request from the corresponding author.

## Abbreviations

The following abbreviations are used in this manuscript:

GFRP	Glass fiber-reinforced plastic
GNP	Graphene nanoplatelet
SB	Secondary bonding
CC	Co-curing
